# Development and Clinical Utility of Machine Learning Models for Prediction of Same‐Day Discharge in Outpatient Hip and Knee Replacement: A Prognostic Study

**DOI:** 10.1111/aas.70318

**Published:** 2026-08-02

**Authors:** Christoffer C. Jørgensen, Jakob B. Frederiksen, Henrik Kehlet, Martin Lindberg‐Larsen, Kirill Gromov, Claus Varnum, Thomas Jakobsen, Manuel Josef Bieder, Mikkel Rathsach Andersen, Søren Overgaard, Torben B. Hansen, Troels Petersen

**Affiliations:** ^1^ Department of Anaesthesia and Intensive Care Hospital of Northern Zealand Hillerød Denmark; ^2^ The Centre for Fast‐Track Hip and Knee Replacement Copenhagen Denmark; ^3^ Faculty of Health Care Science, Institute of Clinical Medicine Copenhagen University Copenhagen Denmark; ^4^ Faculty of Science, The Niels Bohr Institute Copenhagen University Copenhagen Denmark; ^5^ Section of Surgical Pathophysiology, Rigshospitalet Copenhagen University Copenhagen Denmark; ^6^ Denmark Department of Orthopaedic Surgery and Traumatology Odense University Hospital Svendborg Svendborg Denmark; ^7^ Department of Orthopaedic Surgery Hvidovre University Hospital Hvidovre Denmark; ^8^ Department of Orthopaedic Surgery Lillebælt Hospital, Vejle, University Hospital of Southern Denmark Vejle Denmark; ^9^ Department of Orthopaedic Surgery Aalborg University Hospital Farsø Denmark; ^10^ Department of Orthopaedic Surgery Næstved Hospital Næstved Denmark; ^11^ Department of Orthopaedic Surgery Herlev Gentofte Hospital Gentofte Denmark; ^12^ Department of Orthopaedic Surgery Bispebjerg University Hospital Bispebjerg Denmark; ^13^ Department of Orthopaedic Surgery Goedstrup Hospital Goedstrup Denmark

**Keywords:** arthroplasty, artificial intelligence, day surgery, enhanced recovery after surgery, knee, machine‐learning algorithms, predictive learning models, replacement, total hip arthroplasty

## Abstract

**Background:**

Hip and knee replacement are common procedures with an increasing focus on same‐day surgery. However, capacity constraints limit the number of eligible patients actually being scheduled for same‐day discharge, calling for further selection of those with the highest likelihood of same‐day discharge.

**Methods:**

A prognostic study from September 2022 to April 2024 aiming to develop and evaluate three machine learning models of increasing complexity for prediction of successful same‐day discharge after hip and knee replacement. Data was collected from six Danish departments with similar same‐day surgery protocols and same‐day surgery eligibility was according to predefined clinical criteria. The models were evaluated using receiver operating characteristic and clinical utility curves depicting the potential increase in same‐day discharge at different same‐day surgery capacities.

**Results:**

Of 5387 eligible patients, 4466 (82.9%) were scheduled for same‐day surgery. Of these, 3085 (69.1%) achieved same‐day discharge and 1381 (30.9%) were admitted. The remaining 921 (17.1%) were planned as in‐patients. The area under the receiver operating curve showed poor but marginally increasing predictive ability (0.586, 0.602, and 0.603, respectively). Mean probability for same‐day discharge in scheduled same‐day patients was significantly increased in discharged vs. admitted patients (69.90% SD: 7.6 vs. 67.03 SD: 8.3 *p* < 0.001), but not in admitted same‐day versus planned in‐patients (67.52% SD: 7.5 *p* = 0.14). The potential increase in same‐day discharge at the current same‐day surgical capacity of 82.9% of procedures was 2.2% but increased with lower capacities.

**Conclusions:**

Machine learning based prognostic probability scores for planning same‐day hip and knee replacement in pre‐selected eligible patients did not provide relevant potential increases in same‐day discharge rates.

**Editorial Comment:**

This study assessed if an advanced model using routinely available clinical data could confidently predict whether of not cases planned for same‐day hip or knee arthroplasty would be successfully discharged as planned. Data from multiple collaborating fast‐track surgical centers in Denmark contributed to the model. The advanced model here based on the available clinical data did not perform clearly better than other simpler predictive models that have already been reported.

## 1 Introduction

Hip and knee replacement are common surgeries with an expected increase in annual number of procedures due to future demographical changes [1]. This represents a considerable burden on already strained healthcare systems with limited resources and personnel. Through the last decades, fast‐track arthroplasty with a focus on optimisation of perioperative care including mobilization within hours postoperatively, multimodal opioid‐sparing analgesia, avoidance of drains and catheters, and improved logistics, has facilitated early recovery and reduced postoperative morbidity resulting in reductions in length of stay to a median of 1 day [2]. Recently, there has been an increasing focus on same‐day discharge in hip and knee replacement, but with a lack of data from non‐selected patients within socialized health care systems. Data from within a well‐established fast‐track hip and knee replacement collaboration found a same‐day discharge rate of about 65% of planned same‐day procedures [3]. However, only 85% of the eligible patients were included in the same‐day pathway due to restricted capacity [3]. Currently, the choice of which of the eligible patients are to be planned for same‐day surgical procedures is often left to staff selecting from a pool of patients who, apart from being considered overall eligible for safe same‐day discharge, may have other different characteristics potentially affecting the likelihood of same‐day discharge. This may be frustrating for patients who expect to go home, and costly for the hospital due to the need for bed and personnel allocation. As there may be a surplus of patients eligible for same‐day discharge, it is of common interest for both patients and society to predict and optimize the likelihood of same‐day discharge in patients scheduled for same‐day surgical pathways.

Predictive tools for stratification of postoperative complications based on logistical regression analyses are relatively common [4]. Recently there has been an increasing interest in unlocking the potential of machine learning models within anaesthesiology [5], despite that logistic regression models have only slightly inferior accuracy [6]. However, machine learning models may provide additional advantages, including handling of large data volumes from various sources and improving accessibility by being more easily integrated within existing health‐care data systems [5]. Currently, machine learning models have been used for predicting surgical outcomes, length of stay, and complications [6, 7, 8, 9]. However, while some studies have used machine learning models to identify predictors for same‐day discharge after major joint replacement [7, 10], none have investigated a well‐defined population of clinically eligible patients planned for same‐day discharge to predict the probability of same‐day discharge. One of the aims of Fast‐track Center for Hip and Knee Replacement collaboration was to investigate the benefits of machine learning algorithms for predicting same‐day discharge [11]. Here we present a study which developed, validated and evaluated the clinical utility of three machine learning models of increasing complexity, using a combination of pro‐ and retrospective data from established fast‐track centers within a socialized healthcare setting.

## 2 Methods

In September 2022, the Center for Fast‐track Hip and Knee Replacement Collaboration established a prospective database including detailed patient demographics such as preoperative medication, frailty and function and with detailed follow‐up through a mix of patient reported questionnaires and retrospective review of electronic healthcare records. This was done with the overall aim of investigating the feasibility, safety, and socioeconomic aspects of same‐day discharge after hip and knee arthroplasty and development of a prediction model for same‐day discharge [11]. The database was constructed to maximize the potential of machine learning algorithms by including > 70 clinical items with a preference for detailed continuous data, based on previous experience [6]. Data are collected from eight Danish replacement centers from all five Danish Healthcare Regions within the Danish Public Healthcare system. All departments have established fast‐track and same‐day discharge pathways with predefined eligibility criteria [11] (Supporting Information Content 1) and encompass about 40% of the annual number of hip and knee replacements in Denmark. Patients are discharged from a regular ward regardless of being scheduled for same‐day surgery and all are treated according to the same evidence based fast‐track principles as standard of care. These include early mobilization, multimodal opioid‐sparing analgesia, preoperative high‐dose glucocorticoids, functional discharge criteria and discharge to own home [11].

**FIGURE 1:**
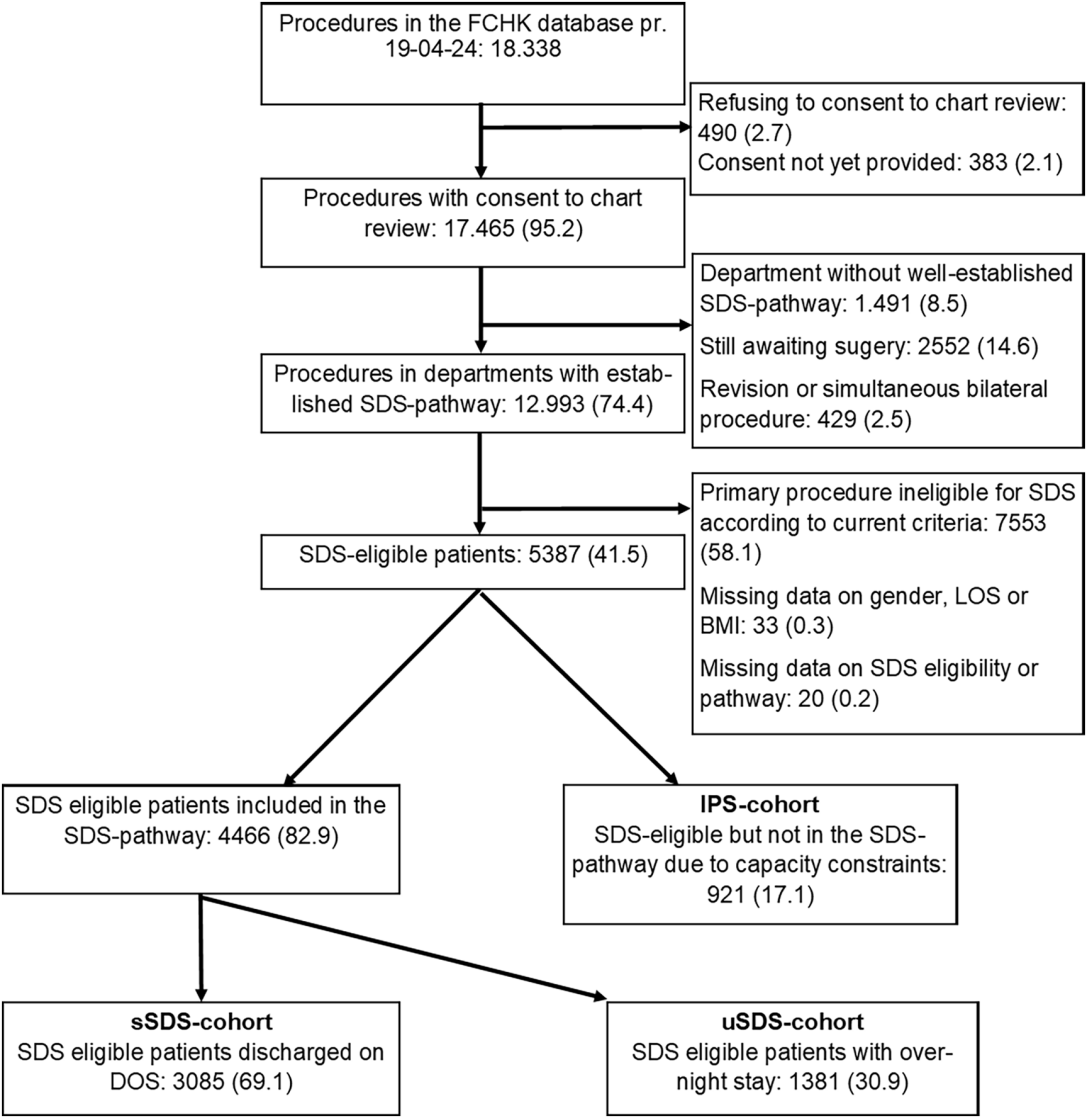
Diagram of the study population. FCHK, Fast‐track Center for Hip and Knee Replacement; LOS, length of hospital stay; BMI, body mass index; SDS, same‐day surgery; sSDS, successful same‐day surgery; uSDS, unsuccesfull same‐day surgery; IPS, planned in‐patient surgery.

For the present study we retrospectively identified all patients eligible for same‐day surgery between September 5, 2022 and April 19, 2024. To minimize potential implementation bias, we excluded two departments without established same‐day surgical pathways from the study start (Figure 1).

### 2.1 Data Collection

Preoperative data were collected prospectively through patient‐reported questionnaires and electronic health‐care records. For Pain Catastrophizing Scale (PCS) scores, patients completed the Danish validated PCS questionnaires, while Clinical Frailty Scale was evaluated by either the surgeon or attending nurse staff. All clinical data were collected by trained personnel and included evaluation of eligibility for same‐day discharge according to predefined patient and procedure related criteria, for example, unilateral primary procedure with scheduled start of surgery before 1 o'clock in the afternoon [11]. Postoperatively, all patients eligible for same‐day discharge were evaluated with regards to whether having been scheduled for same‐day surgery and having same‐day discharge. In case of overnight admission, the cause for staying in hospital was retrospectively recorded according to predefined criteria. Furthermore, any patients who spent more than two nights in hospital during primary admission, were readmitted, or died within 90‐days after surgery also had their medical records retrospectively reviewed.

### 2.2 Outcomes

The primary outcome was the ability to predict likelihood of successful discharge on the day of surgery (sSDS), using three machine‐learning models of increasing complexity.

Secondarily, we investigated differences in the probability distributions in patients with sSDS, in patients needing admission despite planned same‐day discharge (unsuccessful same‐day surgery [uSDS]), and in patients eligible for same‐day surgery but with planned in‐patient surgery (IPS). Finally, we evaluated the potential effect on sSDS rates if using the models when planning which patients were to be scheduled for same‐day surgical procedures compared to current scheduling practice.

### 2.3 Pre‐Study Power Analysis

No pre‐study protocol was prepared, but a pre‐study power analysis for prediction models for binary outcomes was performed using a margin of error less than 5% and with a fraction of same‐day discharge of 65% [3]. This yielded the need for a sample size of (1.96/0.05)^2^ × 0.65 × (1 − 0.65) = 350 [12]. At the time of analysis, a sample size of 4466 patients was available, ensuring the study reached the desired margin of error.

### 2.4 Analysis

The models were constructed to distinguish between the sSDS and uSDS groups by identifying differences in preoperative patient or procedure related characteristics. Once trained, the model can predict the probability of achieving same‐day discharge. To optimize cost–benefit with regards to clinical implementation (model performance vs. complexity) we designed and evaluated three different models with 16 (Basic model), 70 (Common model), and 81 (Complete model) variables, that fell into 11, 23, and 34 clinical groupings, respectively (Table 1). We deliberately excluded department and date of surgery from the input variables to improve generalizability of the model across all departments. To ensure that the variables in the dataset did not involuntarily contain information about dates and the department of surgery, we attempted to predict these using the included variables. We found that missing values for PCS and a CRP level of 4 were related with two departments. To mitigate this, we acquired the missing PCS values and clustered CRP using a minimal value of 4 (Supporting Information Content 2). Model development was made in Python (version 3.10.16) using boosted decision trees (BDTs) implemented via the XGBoost algorithm (version 2.1.1) [13]. The dataset was partitioned using *K*‐fold cross‐validation (K = 20) with a fixed random seed to ensure reproducibility. Within each fold, the data were subdivided into training (75%), validation (10%), calibration (10%), and test (5%) sets. By aggregating predictions across all test sets from each fold, we derived out‐of‐fold predictive estimates for the entire dataset. To mitigate overfitting, early stopping was employed based on performance on the validation set, with a threshold of 25 rounds. Model calibration was subsequently performed using Platt scaling [14] in the Calibrated Classifier CV framework implementation in SciKit‐Learn (version 1.5.2) based on the calibration subset to adjust the predicted outputs, thereby turning BDT output scores into probabilities for identifying patients classified as sSDS. Hyperparameter tuning was done using Optuna (version 4.2.1) [15]. Computational analyses also incorporated NumPy (version 1.26.4) and Pandas (version 2.2.3).

**TABLE 1:** Model variables.

	sSDS (*n*: 3138)	uSDS (*n*: 1381)	IPS (*n*: 921)
*Included in all models*			
Age	66 (59–72)	67 (59–73)	68 (61–74)
Females	1572 (51.0)	549 (39.8)	436 (47.3)
Procedure			
THA	1140 (37.0)	591 (42.8)	387 (41.5)
TKA	1003 (32.5)	533 (38.6)	338 (36.6)
Lateral UKA	39 (1.3)	12 (0.8)	6 (0.7)
Medial UKA	878 (28.4)	240 (17.4)	187 (20.3)
Patellofemoral UKA	25 (0.8)	5 (0.4)	8 (0.9)
BMI	28.0 (25.2–31.2)	27.7 (24.9–31.2)	28.1 (25.1–31.3)
Missing	27 (0.9)	8 (0.6)	9 (1.0)
Use of walking aids			
None	2767 (89.2)	1211 (88.0)	795 (86.3)
Cane or crutches	277 (9.1)	154 (11.2)	105 (11.4)
Walker	39 (1.4)	27 (2.0)	19 (2.1)
Wheelchair	1 (0.0)	1 (0.1)	1 (0.1)
Electric scooter	8 (0.3)	1 (0.1)	4 (0.4)
Cerebral stroke/haemorrhage			
Yes	150 (4.9)	92 (6.7)	56 (6.4)
No	2873 (93.1)	1263 (91.5)	841 (91.5)
Do not remember	36 (1.2)	16 (1.2)	8 (1.0)
Missing	26 (0.8)	10 (0.6)	16 (1.1)
> 1 Fall within 3 months			
Yes	115 (3.8)	51 (3.7)	40 (4.4)
No	2906 (94.2)	1306 (94.6)	858 (93.5)
Do not remember	26 (0.8)	15 (1.1)	7 (0.9)
Missing	38 (1.2)	9 (0.6)	16 (1.2)
Diabetes			
No diabetes	2837 (92.0)	1270 (92.6)	861 (93.5)
Diet‐regulated diabetes	14 (0.4)	10 (0.8)	5 (0.5)
NIDDM	191 (6.1)	84 (6.2)	41 (4.5)
IDDM	11 (0.4)	5 (0.4)	1 (0.1)
Missing	0 (0.0)	0 (0.0)	0 (0.0)
Number of prescribed drugs	3 (2–6)	4 (2–6)	4 (2–7)
Missing	144 (4.7)	53 (3.8)	35 (3.8)
*Included in the “Common” and “Complete” models only*			
Clinical Frailty Scale	2 (2–3)	2 (2–3)	2 (2–3)
Missing	2 (0.1)	0 (0.0)	1 (0.1)
Hemoglobin (mmol/mL)	8.8 (8.3–9.3)	8.7 (8.2–9.2)	8.8 (8.3–9.3)
Missing	5 (0.2)	2 (0.1)	0 (0.0)
Transferrin saturation (%)	27 (21–33)	26 (20–33)	27 (21–34)
Missing^a^	1424 (46.2)	706 (51.1)	432 (46.9)
C‐reactive protein (mg/L)^b^	4 (4–4)	4 (4–4)	4 (4–4)
Missing	41 (1.3)	23 (1.7)	12 (1.3)
Creatinine (μmol/L)	72 (63–83)	70 (61–81)	71 (62–82)
Missing	7 (0.2)	5 (0.4)	2 (0.2)
GFR (mL/min)	87 (76–90)	86 (75–90)	87 (76–90)
Missing	9 (0.3)	5 (0.4)	2 (0.2)
Pain Catastrophizing Scale	15 (8–24)	16 (7–25)	14 (7–23)
Missing	994 (32.2)	342 (24.8)	166 (18.0)
Medically treated pulmonary disease			
None	2663 (86.3)	1151 (86.7)	796 (86.4)
COPD	123 (4.0)	47 (3.5)	28 (3.0)
Asthma	217 (7.0)	100 (7.5)	78 (8.5)
Pulmonary fibrosis	1 (0.0)	0 (0.0)	0 (0.0)
Other pulmonary disease	1 (0.0)	3 (0.2)	1 (0.1)
Missing	0 (0.0)	0 (0.0)	0 (0.0)
Antidiabetic medication			
None	2683 (87.0)	1187 (86.0)	833 (90.4)
Metformin	167 (5.4)	70 (5.1)	34 (3.7)
Sulfonylureas	3 (0.1)	6 (0.4)	1 (0.1)
DPP‐4 inhibitors	10 (0.3)	8 (0.6)	5 (0.5)
GLP‐1 RA	68 (2.2)	18 (1.3)	16 (1.7)
SGLT2‐antagonists (diabetes indication)	65 (2.1)	18 (1.3)	14 (1.5)
Basal insulin	8 (0.3)	2 (0.1)	1 (0.1)
Rapid‐acting insulin	2 (0.1)	2 (0.1)	0 (0.0)
Mixed‐acting insulin	0 (0.0)	0 (0.0)	0 (0.0)
Cardiac medication			
None	1475 (47.8)	680 (49.2)	430 (46.7)
Diuretics	560 (18.2)	285 (20.6)	189 (20.5)
Betablockers	304 (10.0)	138 (10.0)	105 (11.4)
Ca^2+^ antagonists	665 (21.6)	282 (20.4)	198 (21.5)
ANGII/ACE‐antagonists	1088 (35.3)	463 (33.5)	338 (36.7)
Other antihypertensives	35 (1.1)	11 (0.8)	11 (1.2)
Nitro‐glycerine	49 (1.6)	18 (1.3)	17 (1.8)
Other anti‐ischemic drugs	6 (0.2)	0 (0.0)	0 (0.0)
Other antiarrhythmics	7 (0.2)	10 (0.7)	7 (0.8)
SGLT2‐antagonists (cardiac indication)	3 (0.1)	6 (0.4)	1 (0.1)
Missing	0 (0.0)	0 (0.0)	0 (0.0)
Psychotropic drugs			
None	2714 (88.0)	1223 (88.6)	806 (87.5)
SSRI	101 (3.3)	57 (4.1)	38 (4.1)
NARI	0 (0.0)	0 (0.0)	0 (0.0)
SNRI	46 (1.5)	18 (1.3)	18 (2.0)
Tricyclic antidepressants	14 (0.5)	2 (0.1)	3 (0.3)
MAO‐inhibitors	0 (0.0)	1 (0.1)	0 (0.0)
NaSSA	35 (1.1)	16 (1.1)	16 (1.7)
Benzodiazepine	22 (0.7)	10 (0.8)	8 (0.9)
Antipsychotics	28 (0.9)	10 (0.8)	4 (0.4)
Neuroleptics	6 (0.2)	5 (0.4)	2 (0.2)
Other	28 (0.9)	13 (0.9)	10 (1.1)
Missing	0 (0.0)	0 (0.0)	0 (0.0)
Analgesics	1555 (50.4)		
None	1109 (36.0)	678 (49.1)	467 (50.7)
Acetaminophen	460 (14.9)	545 (39.5)	333 (36.2)
NSAIDs	216 (7.0)	217 (15.7)	154
Opioids	150 (4.9)	110 (8.0)	74
Neuroleptics	13 (0.4)	51 (3.7)	35
Other	0 (0.0)	6 (0.4)	5
Missing	0 (0.0)	0 (0.0)	0 (0.0)
Anticoagulants			
None	2457 (79.6)	1081 (78.3)	708 (76.9)
Acetylsalicylic acid	225 (7.3)	100 (7.2)	71 (7.7)
ADP‐antagonists	112 (3.6)	65 (4.7)	36 (3.9)
Dipyridamole	2 (0.1)	1 (0.1)	0 (0.0)
Vitamin‐K antagonists	11 (0.4)	6 (0.4)	6 (0.7)
DOAC	142 (4.6)	78 (5.7)	66 (7.2)
Missing	0 (0.0)	0 (0.0)	0 (0.0)
*Included only in the “Complete” model*			
Joint of surgery			
Hip	1141 (37.0)	591 (46.8)	383 (41.6)
Knee	1944 (63.0)	790 (57.2)	538 (58.4)
Missing	0 (0.0)	0 (0.0)	0 (0.0)
Height (cm)	173 (167–180)	172 (166–179)	173 (167–179)
Missing	20 (0.6)	8 (0.5)	9 (1.0)
Weight (kg)	85 (74–96)	84 (72–95)	85 (74–96)
Missing	26 (0.8)	7 (0.5)	9 (1.0)
Prescribed insulin	26 (0.8)	10 (0.7)	9 (1.0)
Missing	112 (3.6)	38 (2.8)	22 (2.4)
Alcohol > 2 units/day	463 (15.0)	195 (14.1)	134 (14.5)
Missing	26 (0.8)	10 (0.7)	10 (1.)
Habitation			
Living in own home	3041 (98.6)	1365 (98.8)	900 (97.7)
Living in nursing home	7 (0.2)	3 (0.3)	5 (0.5)
Missing	37 (1.2)	13 (0.9)	16 (1.7)
Social status			
Living alone	336 (10.9)	163 (11.8)	128 (13.9)
Living with others	2717 (88.1)	1202 (87.0)	784 (85.1)
Missing	32 (1.0)	16 (1.2)	9 (1.0)
Home care^c^	24 (0.8)	12 (0.9)	13 (1.4)
Missing	32 (1.0)	14 (1.0)	9 (1.0)
Employment			
Employed	1412 (43.6)	553 (40.0)	341 (37.0)
Unemployed	1673 (54.2)	802 (58.1)	553 (60.0)
Missing	67 (2.2)	26 (1.9)	27 (3.0)
Type of employment^d^			
Office work full time	469 (15.2)	208 (37.6)	117 (12.8)
Office work part time	208 (6.7)	90 (16.3)	56 (6.1)
Physical work full time	453 (14.7)	157 (28.4)	117 (12.8)
Physical work part time	213 (6.9)	89 (16.1)	57 (6.3)
Missing	69 (4.9)	9 (2.6)	0 (0.0)
Reason for unemployment^e^			
Retired	1560 (50.6)	752 (93.8)	520 (94.1)
Sick leave	67 (2.2)	32 (4.0)	20 (3.6)
Between jobs	24 (0.8)	9 (1.1)	5 (0.9)
Missing	22 (1.3)	9 (1.1)	8 (1.4)

*Note:* Numbers are *n* (%) or median (IQR). Totals and percentages may exceed the total number of procedures and 100% due to multicategory variables.

Abbreviations: ACE, angiotensin‐converting enzyme; ADP, adenosine diphosphate; ANG, angiotensin; BMI, body mass index; COPD, chronic obstructive pulmonary disease; CRP, C‐reactive protein; DOAC, dual oral anticoagulants; DPP‐4, dipeptidyl peptidase‐4; GFR, glomerular filtration ratio; GLP‐1 RA, glucagon‐like peptide‐1 receptor inhibitors; IDDM, insulin‐dependent diabetes mellitus; IPS, planned inpatient surgery; MAO, monoamine oxidase; NARI, noradrenaline reuptake inhibitor; NaSSA, noradrenergic and specific serotonergic antidepressants; NIDDM, non‐insulin‐dependent diabetes mellitus; NSAID, nonsteroidal anti‐inflammatory drugs; SGLT2, sodium‐glucose cotransporter 2 inhibitor; SNRI, serotonin–noradrenaline reuptake inhibitor; SSRI, selective serotonin reuptake inhibitor; sSDS, successful same‐day surgery; THA, total hip arthroplasty; TKA, total knee arthroplasty; UKA, unilateral knee arthroplasty; uSDS, unsuccessful same‐day surgery.

^a^ Transferrin saturation was only mandatory in patients with a hemoglobin of < 8 mmol/mL.

^b^ CRP was binned for all values < 4 mg/L.

^c^ Registered for “living in own home only.”

^d^ Registered for “Employed only.”

^e^ Registered for “Unemployed only.”

Parameters subject to optimization included maximum tree depth, learning rate, gamma, and L1/L2 regularization coefficients. Optimal configurations were selected by minimizing the Binary Cross Entropy (Supporting Information Content 3). Model performance was assessed through the area under the receiver operating curve (AUC). Calibration improved model reliability as evidenced by the reduced Mean Squared Error values (Supporting Information Content 4). Comparison of hyperparameters was largely similar between models. To quantify feature importance, SHapley Additive exPlanations (SHAP) values were computed using the SHAP package (version 0.46.0) [16]. Finally, we evaluated clinical utility through plots of sSDS rates according to the probability scores.

### 2.5 Ethical Considerations

This was an observational study which is exempt from approval by the Danish Ethics committee. All patients provided written informed consent to the overall scientific aims of the Fast‐track Center for Hip and Knee Replacement collaboration. Permission to collect, store and process data was acquired from the Region of Southern Denmark (Jnr. 22/39454). The RedCap Database system [17] is approved by the Danish National Dataprotection Agency for use in all Danish Healthcare Regions. The database is registered at ClinicalTrials.gov (NCT05613439).

## 3 Results

**FIGURE 2:**
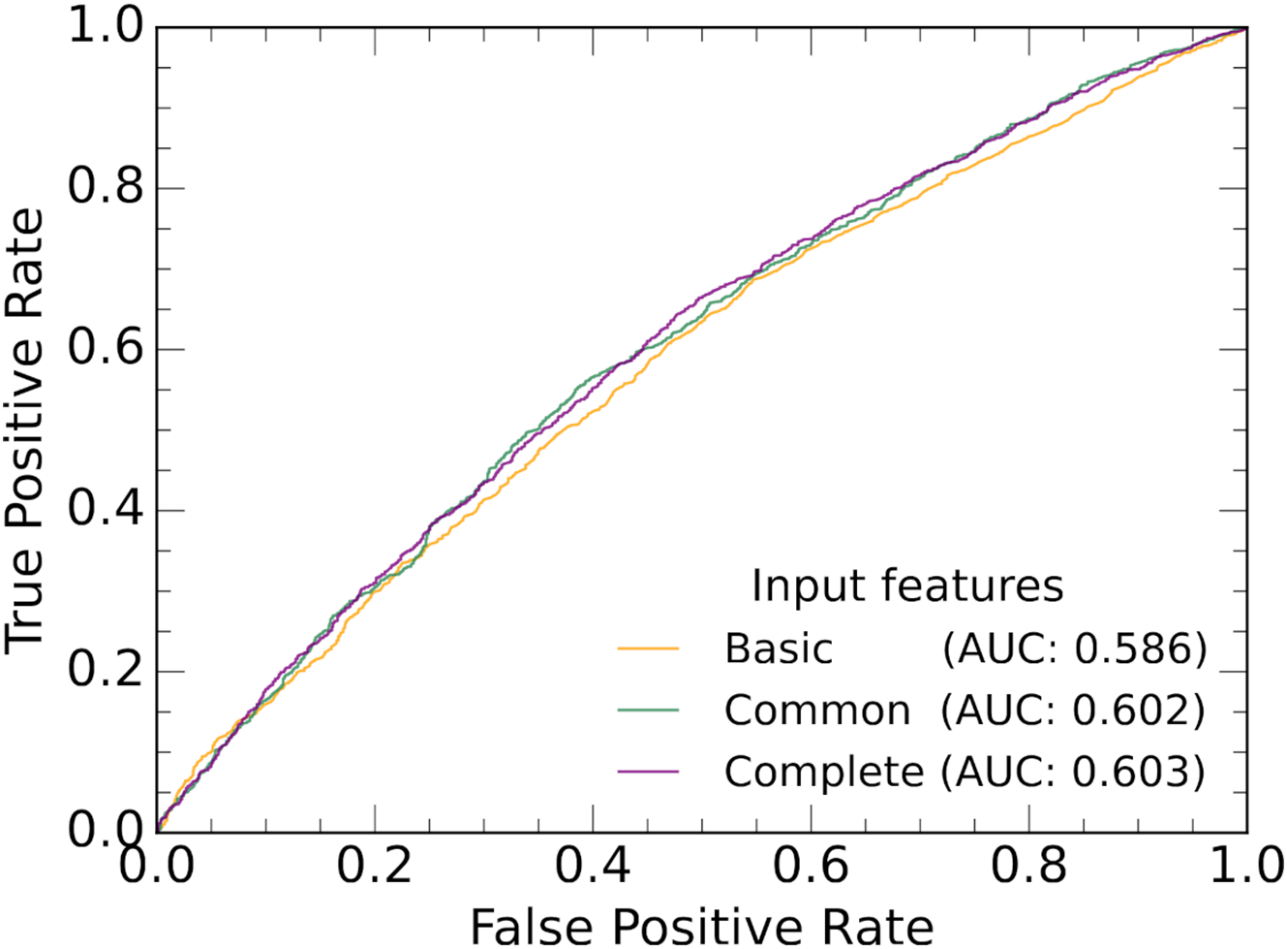
Receiver operating curve of model performance. The area under the curve (AUC) for the three developed models indicates the ability of the model to predict same‐day discharge. The low AUC (poor to fair performance) is not unexpected due to initial clinical selection for same‐day discharge eligibility.

**FIGURE 3:**
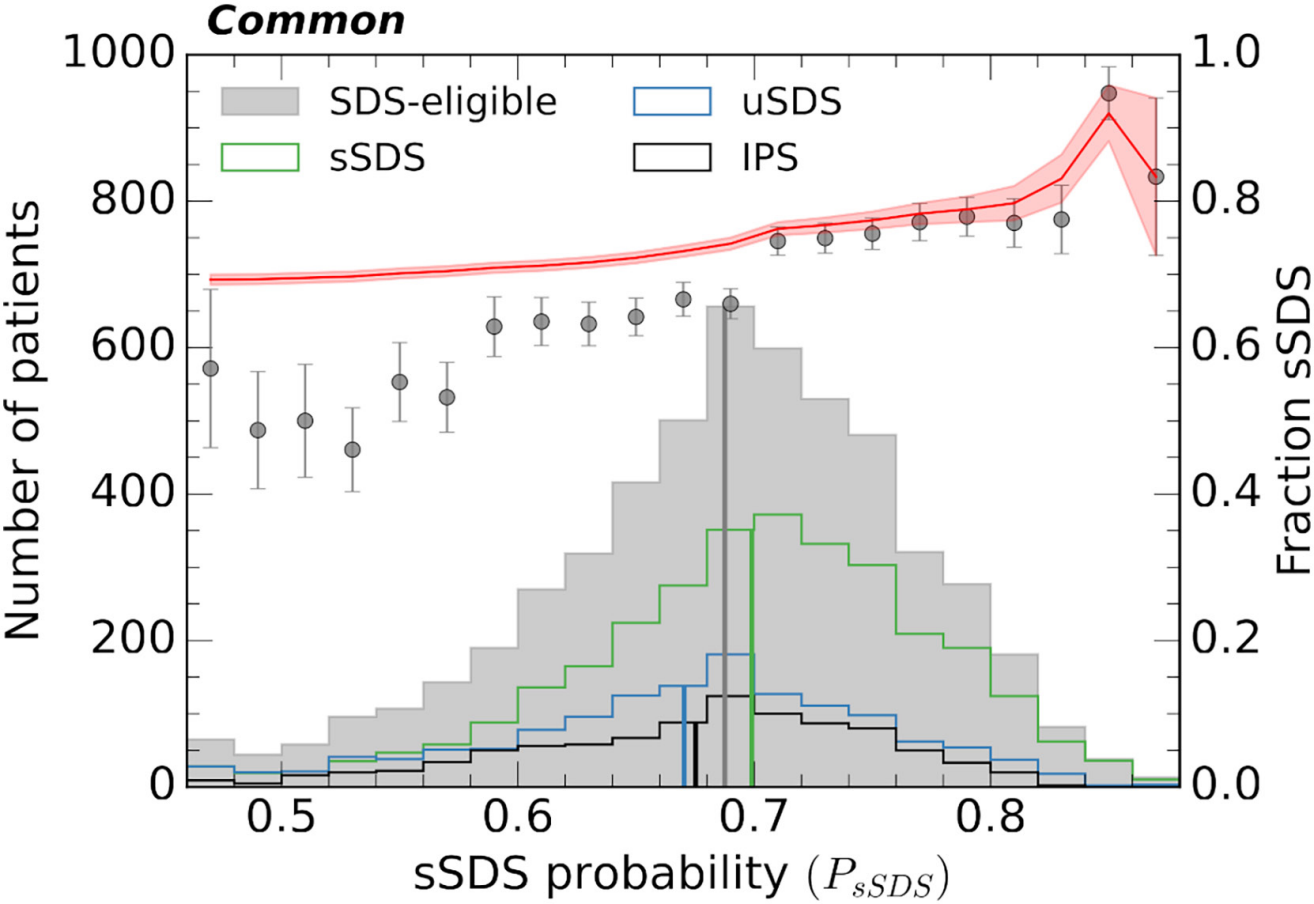
Distribution of probability scores for successful same‐day surgery. Distribution of probability scores for successful same‐day surgery (sSDS) in the sSDS, unsuccessful same‐day surgery (uSDS), and planned in‐patient surgery (IPS) cohorts. Vertical lines indicate mean probability score for each cohort. The histogram (gray) indicates the total number of eligible same‐day surgery (SDS) patients in each bin. Dots with error bars indicate the average fraction (68.2% CI) of sSDS patients in each bin. For example, the bin with a sSDS probability of 0.60–0.62 includes ≈275 patients with an average sSDS fraction ≈65%. The red line indicates the accumulated sSDS proportion (68.2% CI) at each threshold of sSDS. For example, if using a sSDS probability threshold of 0.6 for including patients in the SDS‐pathway, the fraction with sSDS would be ≈72% and include all patients with a probability score of ≥ 0.6.

Of 12,993 procedures, 5387 (41.5%) were eligible for same‐day surgery with 4466 (82.9%) patients included in the same‐day surgical pathway. Of these, 3085 (69.1%) were discharged on day of surgery (sSDS) and 1381 (30.9%) were admitted for a median of 1 day (uSDS). Of the uSDS patients, 56 (4.1%) spent one night in hospital due to late return to the ward or prolonged motor‐blockade after spinal anesthesia and 42 (3.0%) had a length of stay > 2 days. Of the three constructed models, the common model based on 70 variables from 23 groupings, performed equally to the Complete model including all 81 variables from 34 groupings. The AUC values increased marginally but with no additional gain from using the Complete model (Figure 2). The distribution of the probability scores demonstrated major overlaps and minimal differences between the sSDS and uSDS populations (Figure 3). When using the Common model (fewest predictors with slightly improved AUC), the probability scores ranged from 56% to 81%. The mean probability score for the Common model was significantly different between the sSDS and uSDS patients, although the actual difference was limited (69.90% ± 0.14%; SD: 7.6 vs. 67.03% ± 0.22%; SD: 8.3, double‐sided *z*‐test *p* < 0.001). The same applied to the Basic (69.73% ± 0.13%; SD: 7.2 vs. 67.57% ± 0.19%; SD: 7.0, double‐sided *z*‐test *p* < 0.001) and Complete (69.92% ± 0.13%; SD: 7.5 vs. 67.12% ± 0.21%; SD: 8.0, double‐sided *z*‐test *p* < 0.001) models (Supporting Information Content 5 and 6).

In the 921 IPS patients, median LOS was 1 day, 117 (12.7%) were discharged on the day of surgery and 30 (3.3%) had a length of hospital stay > 2 days. All models found the mean probability scores of IPS patients to lie between the means of the sSDS and uSDS patients (Common: 67.52% ± 0.25% SD: 7.5, Basic: 68.27% ± 0.21% SD: 6.4, Complete: 67.59% ± 0.24% SD: 7.2) (Figure 3 and Supporting Information Content 5 and 6). However, the probability score distribution in IPS patients was closest to and statistically indistinguishable from the uSDS patients when using the Common and Complete (*p* = 0.141) but not the Basic model (*p* = 0.013).

### 3.1 Feature Importance

SHAP‐analysis showed good agreement across the three models on feature importance. The Basic model relied on type of procedure, sex, number of prescriptions, age, and BMI, while the Common and Complete models also included CFS, hemoglobin (HB) level, Transferrin saturation (Tsat), and PCS (Supporting Information Content 7). However, regardless of the chosen model, procedure type (mainly any type of unicompartmental knee replacement vs. total hip or knee replacement), and gender remained the most important contributors. This is exemplified by SHAP analysis for individual feature importance in a patient with a high and a low probability score, respectively. The probability score of patient A was positively influenced by having medial UKR, male sex, only one prescription, a BMI of 36.4 and Tsat of 0.34. In contrast, a CSF of 3 and PCS of 19 were the main negative contributors of patient A's final probability score of 80.6% (Supporting Information Content 8). Patient B is a 61‐year‐old female having total knee replacement with a CFS of 3, a PCS of 37, and 8 different prescribed drugs, all which had a major negative influence on her probability score. Despite a BMI of 35.7 and a GFR of 87 contributing positively, patient B's final probability score was only 54.6% (Supporting Information Content 9).

### 3.2 Clinical Utility Analysis

**FIGURE 4:**
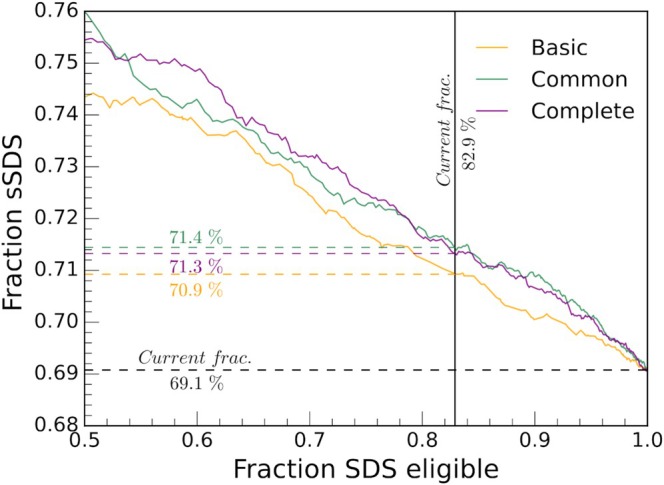
Clinical utility graph of potential increase in same‐day discharge if using the models. Dotted line illustrates current practice with 82.9% of eligible same‐day surgery (SDS) patients being included in the SDS‐pathway and with a success rate of 69.1%. The yellow, green, and purple lines depict success rate if using the Basic, Common, or Complete model. Thus, an increase in successful same‐day discharge of 2.2% is expected when using the Common model and selecting the patients with the highest predicted probabilities vs. current practice (horizontal dotted black line) at the current SDS capacity of 82.9% (vertical solid black line).

When estimating the potential benefits on success rate by selecting the patients most probable for sSDS for the SDS pathway, the fraction of sSDS patients would increase by 1.8% if using the Basic model and 2.2% if using the Common or Complete model within current SDS capacity of 82.9% (Figure 4). This is equal to a relative increase of 3.18%. However, the benefits of using any model would be further accentuated in departments with fewer available SDS slots. Thus, if SDS capacity only allowed 70% of eligible patients to be scheduled for SDS, the potential increase in sSDS when using the Common model would be 3.76%, corresponding to a relative increase in sSDS patients of 5.44% (Table 2). Notably, there was no additional clinical benefit of using the Complete model (Figure 4).

**TABLE 2:** Impact of planning same‐day surgery stratified by highest probability scores.

	90%	80%	70%	60%	50%
*Actual increase in fraction with same‐day discharge*					
Model					
Basic	0.98	2.20	3.37	4.62	5.34
Common	1.82	2.71	3.76	5.23	6.93
Complete	1.56	2.54	4.12	5.77	6.37
*Relative increase in fraction with same‐day discharge*					
Model					
Basic	1.42	3.19	4.88	6.69	7.74
Common	2.64	3.92	5.44	7.56	10.03
Complete	2.26	3.67	5.96	8.35	9.22

*Note:* Actual and relative increases in same‐day discharge with decreasing same‐day surgical capacities (percentages) and increasing complexity of the machine learning model (Basic, Common, Complete) compared to current the same‐day discharge rate of 69.1%. All numbers are %.

## 4 Discussion

In this study, BDTs were used to predict which eligible same‐day surgery patients were most likely to have same‐day discharge after hip or knee replacement. We developed three different models with gradually increasing complexity, reflecting clinical and demographic data which have previously been found to influence postoperative outcomes and length of hospital stay.

Despite the models being able to discriminate between the sSDS and uSDS cohorts, there was a considerable overlap in probability scores, prohibiting clinically relevant application on an individual level. Although low AUC values were to be expected as a selection process had already been performed prior to constructing the models, clinical relevance was further limited by only minor potential increases in sSDS patients within current capacity constraints. However, our data reflect the average eligibility and success rates across six different departments as we ensured the model could not identify department of surgery. Thus, the accuracy of our model will vary between departments depending on their case mix and may be better suited for institutions with limited same‐day surgical capacity. Interestingly, the mean predicted sSDS probability of the planned in‐patients was similar to the uSDS patients. This could indicate a degree of additional selection when deciding which of the eligible patients were chosen for same‐day surgery of which we were not aware.

We found only a limited benefit of using more complex models compared to the Basic model with only 11 clinical groupings and no advantage of using the model with all 34 clinical groupings (Complete model) compared to the model using 23 clinical groupings (Common model). That the benefits of increasing the number of patient‐related variables did not increase model performance may indicate that lack of same‐day discharge success is often driven by organizational and logistical rather than patient‐related factors. Consequently, there may be a need to differentiate between medical and logistical causes of admission in patients planned for same‐day discharge. Thus, in our cohort about one in 20 of the uSDS patients stayed in hospital due to logistical issues like late return to the ward despite no registered complications or prolonged motor‐block after spinal anaesthesia delaying adequate mobilization. Such issues may not be predicted based on patient‐level variables prior to surgery and necessitate organizational rather than patient‐selection changes (e.g., optimizing work‐flows to avoid surgical delay, type and dosing of local anaesthetic for spinal anaesthesia). In contrast, medical complications or impaired mobilization may be related to patient‐specific features present at the preoperative visit (comorbidities, frailty etc.). Nevertheless, the lack of improvement despite increasing model complexity is discouraging as the inclusion of increasingly large and complex datasets is supposed to be one of the strengths of machine learning models [5].

Our study differs from previous machine‐learning studies in not attempting to predict eligibility or safety, but instead predicting sSDS in well‐defined SDS eligible patients [18, 19, 20, 21]. Thus, previous prediction models have been developed to support clinical decision making [22], by predicting likelihood of surgical complications [23], length of stay [24], and readmissions [25]. Focus has been on primary selection of patients without previous clinical evaluation on eligibility, and often including intraoperative features such as duration of surgery and choice of anesthesia [7, 10, 21]. Our study focuses on potential advantages of a machine learning model for predicting which patients out of a population already deemed eligible according to clinical criteria [11], are most likely to achieve same‐day discharge.

SHAP‐analysis found that UKR was associated with increased probability of sSDS, supporting previous studies demonstrating increased same‐day discharge in patients having UKR [3, 26]. Male sex also increased the probability score compared to females. Whether the gender difference is due to physiological or sociological differences is speculative and could be an area for further research. Preoperative creatinine, Hb and Tsat were also among the nine most important features predicting sSDS probability. That laboratory parameters may be of importance for predicting same‐day discharge was also described in a previous study in unselected patients having total hip replacement [19]. However, it must be considered that we did not standardise the time of preoperative blood samples more specifically than within 30 days prior to surgery which may be a limitation. CFS, PCS, number of prescribed medications and age at surgery were the most important non‐laboratory parameters. Frailty is gaining increasing interest as a preoperative risk factor associated with increased LOS and postoperative morbidity [27]. Interestingly, despite only patients “managing well” or better according to the CFS (CFS ≤ 3) [28] were eligible for SDS, CFS was one of the most important features for predicting sSDS. In contrast, established risk factors such as use of walking aids or living alone were of less importance. This likely reflects the initial patient selection where patients with high CFS or repeated falls were excluded but could also be due to associations beyond the ability of SHAP‐analysis to explain [29].

The strengths of our study include a prospective standardized multicenter same‐day surgical setup with similar eligibility and discharge criteria. Furthermore, data collection was designed to complement machine learning methods based on previous experiences [6]. Also, all data went through a thorough evaluation of input variables to ensure that they did not contain additional information, as exemplified by the need for clustering of CRP levels. Finally, the construction of a percentage‐based scale for likelihood of same‐day discharge and the inclusion of clinical utility curves allows for an easily interpretable model and estimation of potential clinical benefits.

Several limitations may apply to the models. Thus, the predictive power was only poor to fair. Although this is partly explainable due to primary clinical selection on eligibility, there would likely be a benefit from inclusion of additional variables such as exact time of surgery and choice of anesthesia. The availability of individual probability scores for having surgery in the morning or afternoon or with different anesthetic techniques would also increase clinical usefulness. Regarding the applied clinical eligibility criteria for SDS, these are not well defined for hip and knee replacement and may be challenged. Thus, the criteria were based on previously published data on patient related risk‐factors [6, 30] and local logistical considerations. However, other criteria focusing on preoperative function, social network, or medical optimization have also been proposed [31, 32]. In this context, our model is not able to evaluate whether the used eligibility criteria are justified as the models were only trained and tested on patients who were already considered eligible for same‐day surgery pathways. Consequently, the proposed models are unable to consider the characteristics and postoperative outcomes of patients considered ineligible for SDS.

In conclusion, although all three prognostic machine learning models were able to predict the probability of same‐day discharge in pre‐selected eligible patients, the improvement of model accuracy with increasing model complexity was limited. Furthermore, clinical utility was limited, with probability score‐based patient selection being unlikely to result in clinically relevant increases in successful same‐day discharge.

## Author Contributions

Data access and integrity: Christoffer C. Jørgensen and Henrik Kehlet. Responsibility for the accuracy of the data analysis: Christoffer C. Jørgensen, Jakob B. Frederiksen, and Troels Petersen. Concept and design: Christoffer C. Jørgensen, Jakob B. Frederiksen, Henrik Kehlet, and Troels Petersen. Drafting the manuscript: Christoffer C. Jørgensen, Jakob B. Frederiksen, and Troels Petersen. Statistical analysis: Jakob B. Frederiksen and Troels Petersen. Critical review of the manuscript for intellectual content: All authors. Acquisition, analysis, or data interpretation: All authors.

## Funding

The study was funded by a grant from the Novo Nordisk Foundation, Denmark (grant number: NNF21SA0073760) and Candys Foundation, Denmark. The funders of the study had no role in study design, data collection, data analysis, data interpretation, or writing of the manuscript.

## Conflicts of Interest

C.C.J. is a member of the board for the Danish Society of Ambulatory Surgery and reports having received reimbursement for travel expenses and personal speaker's fees from Pharamacosmos outside of the present study. C.V. has received reimbursement of travel expenses from Stryker paid to his institution. S.O. is Editor in Chief of *Acta Orthopaedica* and has received speaker's fees from Heraeus. The other authors declare no conflicts of interest.

## Supporting information


**Supporting Information Content 1:** Inclusion and exclusion criteria for eligibility of planned same‐day discharge.
**Supporting Information Content 2:** Distribution of preoperative C‐Reactive Protein level before and after clustering.
**Supporting Information Content 3:** Comparison of hyperparameters across the three XGBoost models of increasing complexity.
**Supporting Information Content 4:** Calibration plots for all models.
**Supporting Information Content 5:** Distribution of same‐day discharge probabilities for the Basic model.
**Supporting Information Content 6:** Distribution of same‐day discharge probabilities for the Complete model.
**Supporting Information Content 7:** SHapley Additive exPlanations analyses of feature importance.
**Supporting Information Content 8:** SHapley Additive exPlanations analyses: Patient with high same‐day discharge probability.
**Supporting Information Content 9:** Individual SHapley Additive exPlanations analyses: Patient with low same‐day discharge probability.

## Data Availability

All datacode is freely available from Git.Hub. The dataset is not available for sharing in its complete form due to Danish legislation. A modified and deidentified dataset of the development and test population is available from the corresponding author upon request for reasonable academic purposes only.
