# Supplementary material for: Development and Clinical Utility of Machine Learning Models for Prediction of Same‐Day Discharge in Outpatient Hip and Knee Replacement: A Prognostic Study
Source: Acta Anaesthesiol Scand. 2026 Aug 2;70(8):e70318. doi: 10.1111/aas.70318 (PMC13429949; doi:10.1111/aas.70318)
Supplement: Supplementary file 1 — Supporting Information Content 1: Inclusion and exclusion criteria for eligibility of planned same‐day discharge. Supporting Information Content 2: Distribution of preoperative C‐Reactive Protein level before and after clustering. Supporting Information Content 3: Comparison of hyperparameters across the three XGBoost models of increasing complexity. Supporting Information Content 4: Calibration plots for all models. Supporting Information Content 5: Distribution of same‐day discharge probabilities for the Basic model. Supporting Information Content 6: Distribution of same‐day discharge probabilities for the Complete model. Supporting Information Content 7: SHapley Additive exPlanations analyses of feature importance. Supporting Information Content 8: SHapley Additive exPlanations analyses: Patient with high same‐day discharge probability. Supporting Information Content 9: Individual SHapley Additive exPlanations analyses: Patient with low same‐day discharge probability. [file AAS-70-0-s001.docx]

Supplementary Content

[Supplementary Content 1 Inclusion and exclusion criteria for eligibility of planned same-day discharge 1](#_Toc213773694)

[Supplementary Content 2 Distribution of preoperative C-Reactive Protein level before and after clustering. 2](#_Toc213773695)

[Supplementary Content 3 Comparison of hyperparameters across the three XGBoost models of increasing complexity. 3](#_Toc213773696)

[Supplementary Content 4 Calibration plots for all models 4](#_Toc213773697)

[Supplementary Content 5 Distribution of same-day discharge probabilities for the Basic model 5](#_Toc213773698)

[Supplementary Content 6 Distribution of same-day discharge probabilities for the Complete model 6](#_Toc213773699)

[Supplementary Content 7 SHapley Additive exPlanations analyses of feature importance 7](#_Toc213773700)

[Supplementary Content 8 SHapley Additive exPlanations analyses: Patient with high same-day discharge probability. 8](#_Toc213773701)

[Supplementary Content 9 Individual SHapley Additive exPlanations analyses: Patient with low same-day discharge probability. 9](#_Toc213773702)

## Supplementary Content 1 Inclusion and exclusion criteria for eligibility of planned same-day discharge

| **Inclusion criteria** |
| --- |
| Unilateral elective primary total hip replacement, total knee replacement or unicompartmental knee replacement  Age 18-80 years |
| **Exclusion criteria** |
| Acute myocardial infarction, cerebrovascular accident, transient ischemic attack, or coronary atherosclerotic disease within last 3 months  Unstable ischemic heart disease  Ejection fraction < 40%  Glomerular filtration rate < 60 mL/min/1.73 m2  Chronic obstructive pulmonary disease with home oxygen  Insulin-dependent diabetes mellitus  Sleep apnea requiring mechanical treatment  Clinical Frailty Scale ≥ 4 (35)  2 or more falls within last 3 months  Body mass index < 18.5 or > 40  Other^1^  Not interested in discharge on day of surgery^2^ |
| ^1^Included to allow for registering special cases at the discretion of the attending surgeon.  ^2^Removed in most departments after 2022 as same-day discharge became a standard of care. |

## Supplementary Content 2 Distribution of preoperative C-Reactive Protein level before and after clustering.


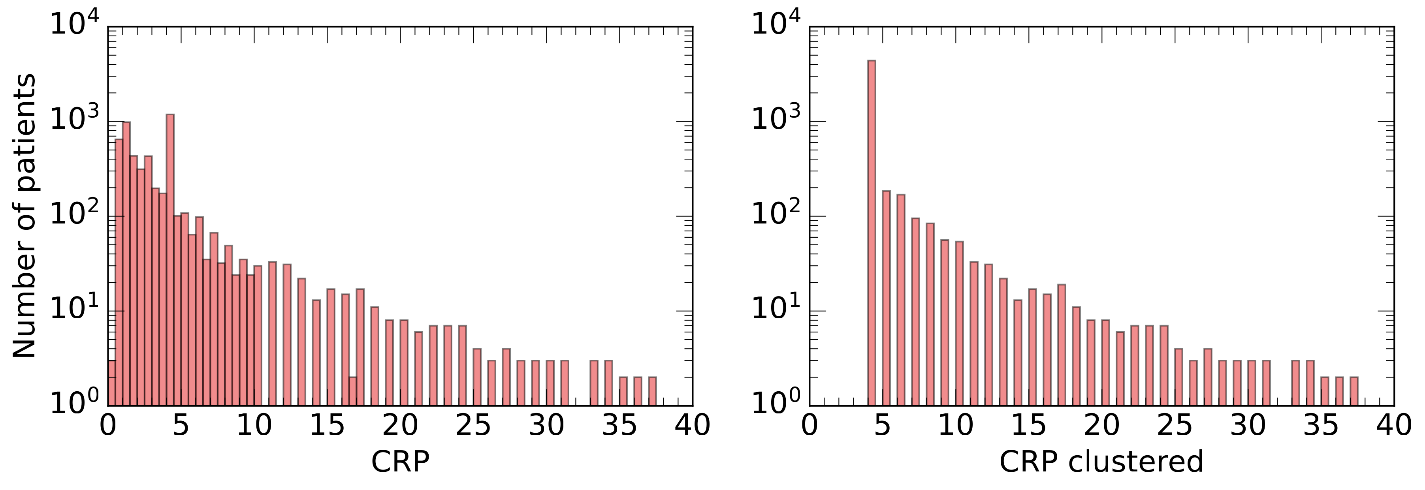
The top at a C-Reactive Protein (CRP) level of 4 was mainly due to one department having a laboratory reporting a CRP of 4 as the lowest threshold. Consequently, to mitigate relation to department clustering was applied with aggregation of all CRP levels ≤4.

## Supplementary Content 3 Comparison of hyperparameters across the three XGBoost models of increasing complexity.

| **Model** | **Max depth** | $\boldsymbol{\eta}$ | $\boldsymbol{\gamma}$ | $\boldsymbol{\alpha}$ | $\boldsymbol{\lambda}$ | **Early stopping rounds** | **N  estimators** |
| --- | --- | --- | --- | --- | --- | --- | --- |
| Basic | 2 | 0.122025 | 2.834783 | 0.991652 | 0.528150 | 25 | 1000 |
| Common | 3 | 0.056207 | 0.287396 | 0.502327 | 0.428999 |  |  |
| Complete | 2 | 0.146756 | 0.813397 | 0.909821 | 0.282067 |  |  |
| Max depth controls tree depth and model complexity; learning rate influences the contribution of each tree to the final prediction; gamma sets the minimum loss reduction required for node splitting; alpha is the L1 regularization term that helps prevent overfitting; and lambda is the L2 regularization parameter that further controls model complexity. The basic model uses minimal features, while the common and all models incorporate progressively more features for prediction. Early stopping rounds and N estimators were kept fixed for all models. | | | | | | | |

## Supplementary Content 4 Calibration plots for all models


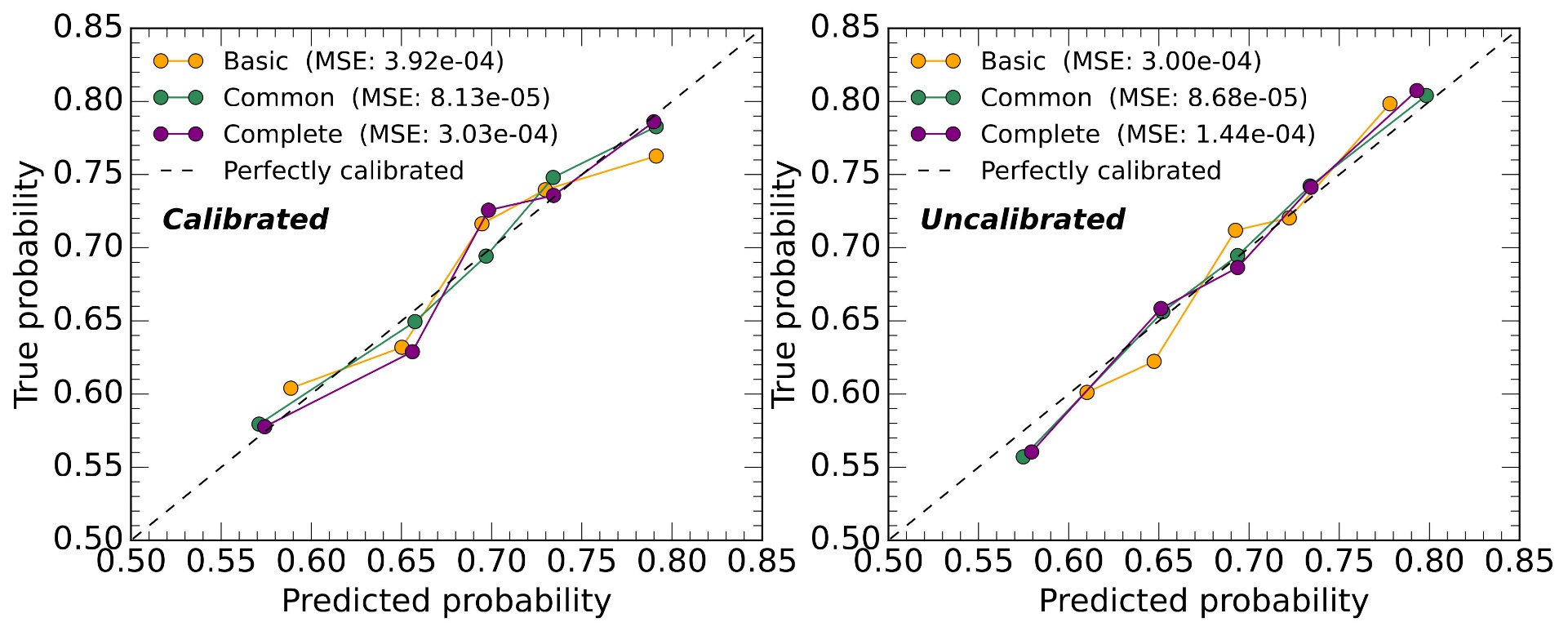


Calibration plots demonstrating improved model reliability as evidenced by the reduced Mean Squared Error (MSE) values for all models.

## Supplementary Content 5 Distribution of same-day discharge probabilities for the Basic model


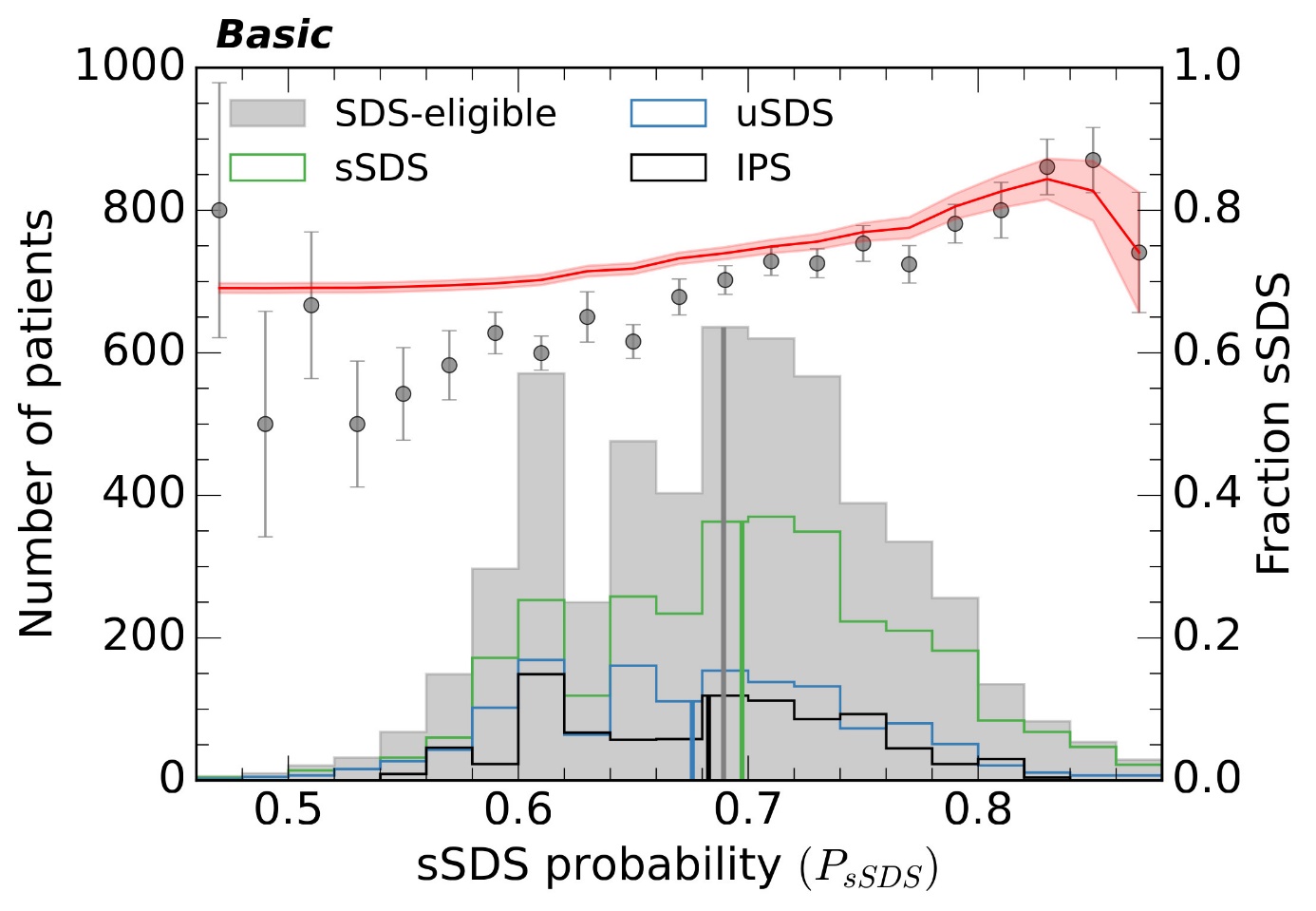


Distribution of probability scores for successful same-day surgery (sSDS) in the sSDS, unsucessfull same-day surgery (uSDS) and planned in-patient surgery (IPS) cohorts. Vertical lines indicate mean probability score for each cohort. The histogram (grey) indicates the total number of eligible same-day surgery (SDS) patients in each bin of. Dots with errorbars indicate the average fraction (68.2%CI) of sSDS patients in each bin. E.g. the bin with a sSDS probability of 0.60-0.62 includes ≈ 550 patients with an average sSDS fraction ≈ 60%. The red line indicates the accumulated sSDS proportion (68.2%CI) at each threshold of sSDS. E.g. if using a sSDS probability threshold of 0.6 for including patients in the SDS-pathway, the fraction with sSDS would be ≈ 72% and include all patients with a probability score of ≥ 0.6.

## Supplementary Content 6 Distribution of same-day discharge probabilities for the Complete model


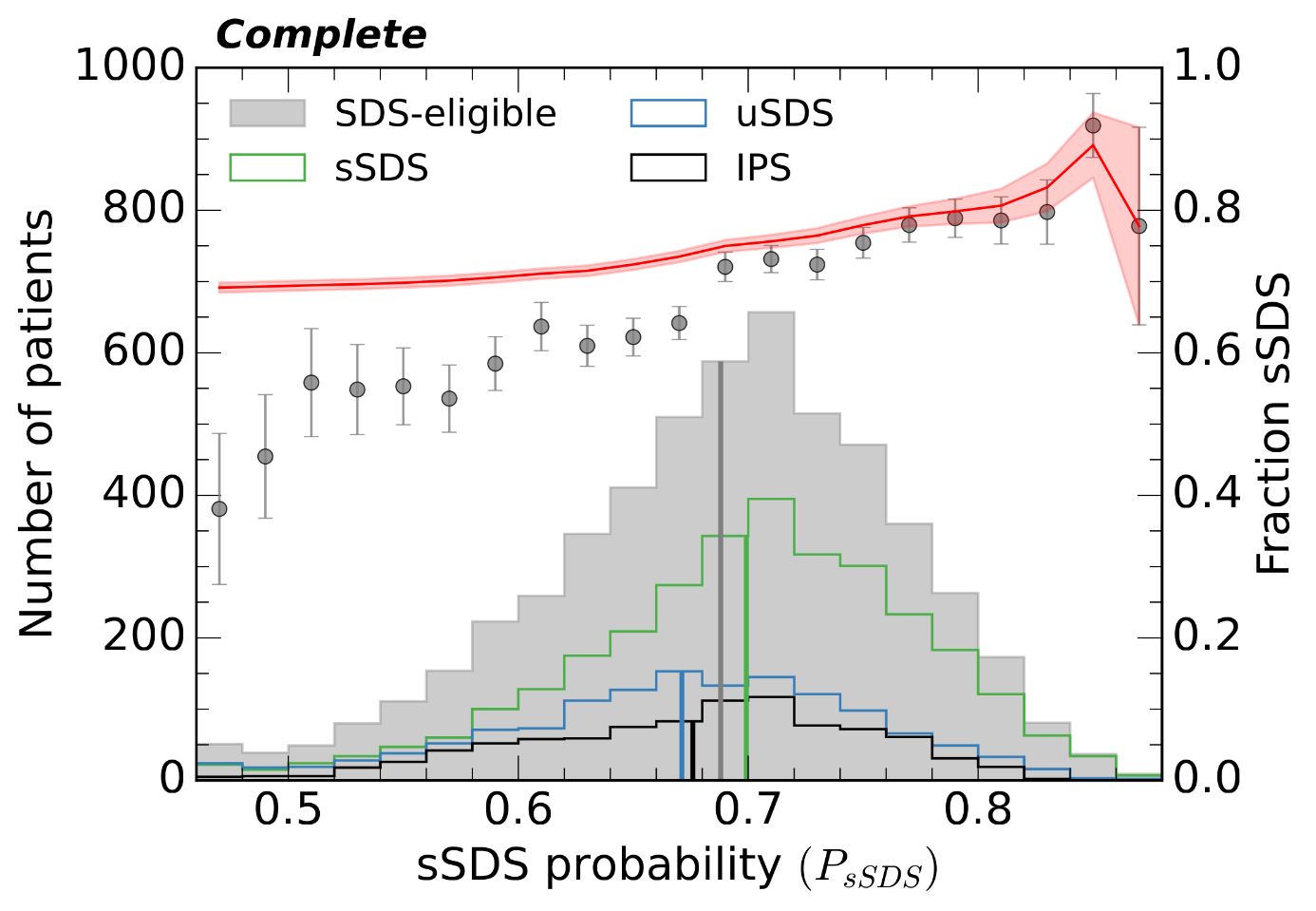


Distribution of probability scores for successful same-day surgery (sSDS) in the sSDS, unsucessfull same-day surgery (uSDS) and planned in-patient surgery (IPS) cohorts. Vertical lines indicate mean probability score for each cohort. The histogram (grey) indicates the total number of eligible same-day surgery (SDS) patients in each bin of. Dots with errorbars indicate the average fraction (68.2%CI) of sSDS patients in each bin. E.g. the bin with a sSDS probabilty of 0.60-0.62 includes ≈ 275 patients with an average sSDS fraction ≈ 65%. The red line indicates the accumulated sSDS proportion (68.2%CI) at each threshold of sSDS. E.g. if using a sSDS probability threshold of 0.6 for including patients in the SDS-pathway, the fraction with sSDS would be ≈ 72% and include all patients with a probability score of ≥ 0.6.

## Supplementary Content 7 SHapley Additive exPlanations analyses of feature importance


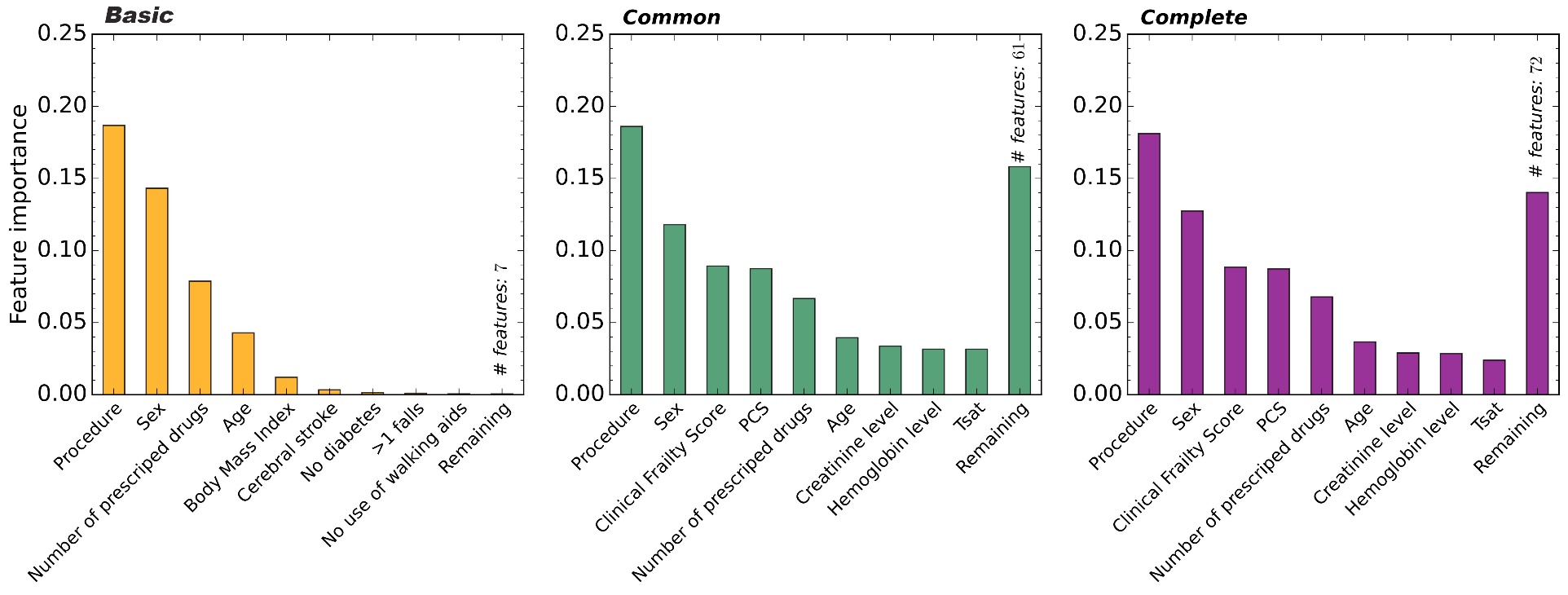
Only the top 9 features are depicted for each model. The number and importance of the remaining features is summarized in the “Remaining” category. PCS: Pain catastrophizing score Tsat: transferrin saturation.

## Supplementary Content 8 SHapley Additive exPlanations analyses: Patient with high same-day discharge probability.


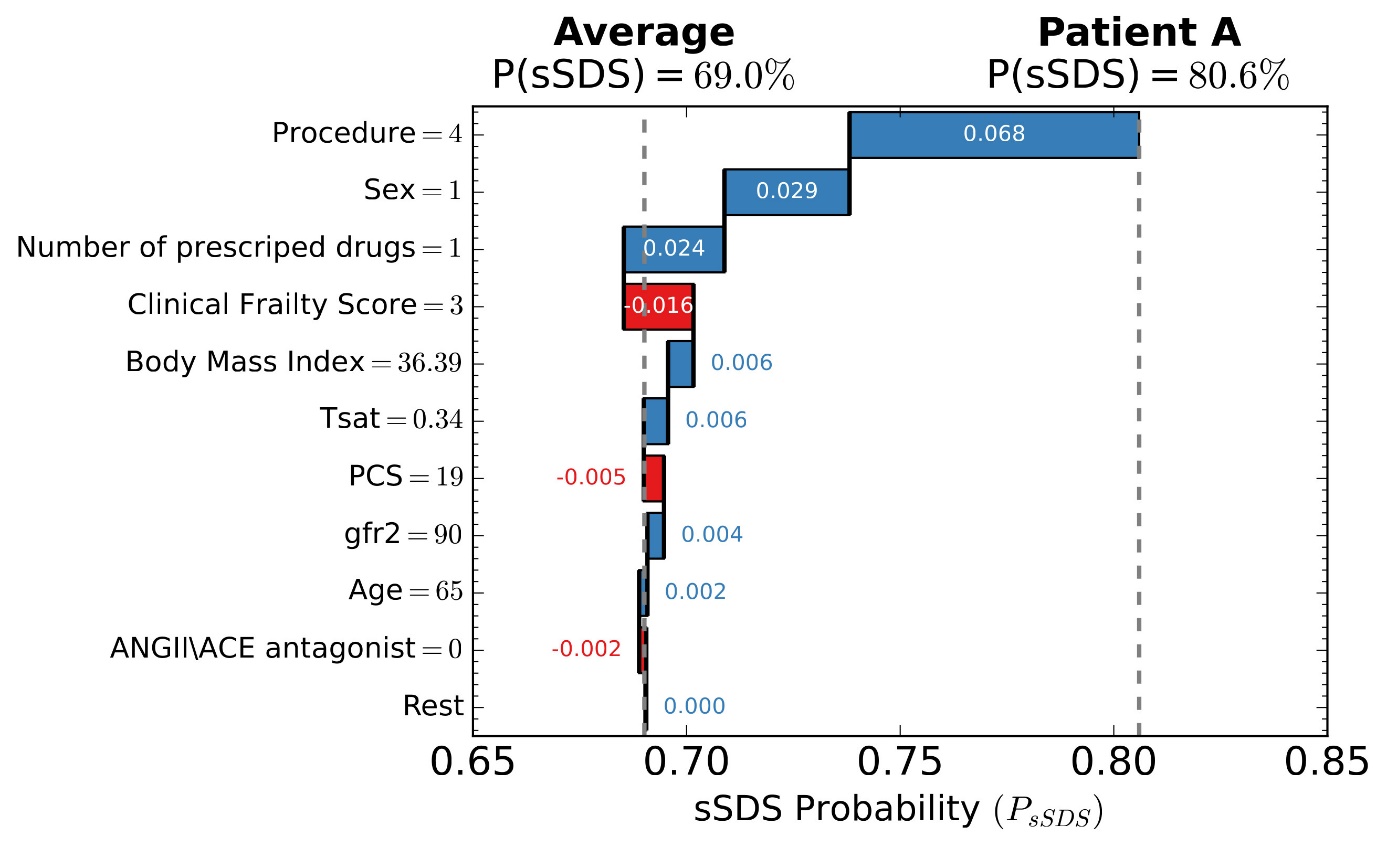


SHAP analysis of feature importances for probability of successful same-day surgery (sSDS) in a patient with a higher-than-average likelihood. Sex= 0 indicates female gender, Procedure 4 indicates a medial unicompartmental knee arthroplasty and no analgesics = 0 indicates that the patient had prescribed analgesic medication. PCS: Pain catastrophizing score, Tsat: Transferrin saturation. Rest= Accumulated influence of remaining variables in the Common model. Dotted lines mark the calculated probability of sSDS in Patient A and the population average, respectively.

## Supplementary Content 9 Individual SHapley Additive exPlanations analyses: Patient with low same-day discharge probability.


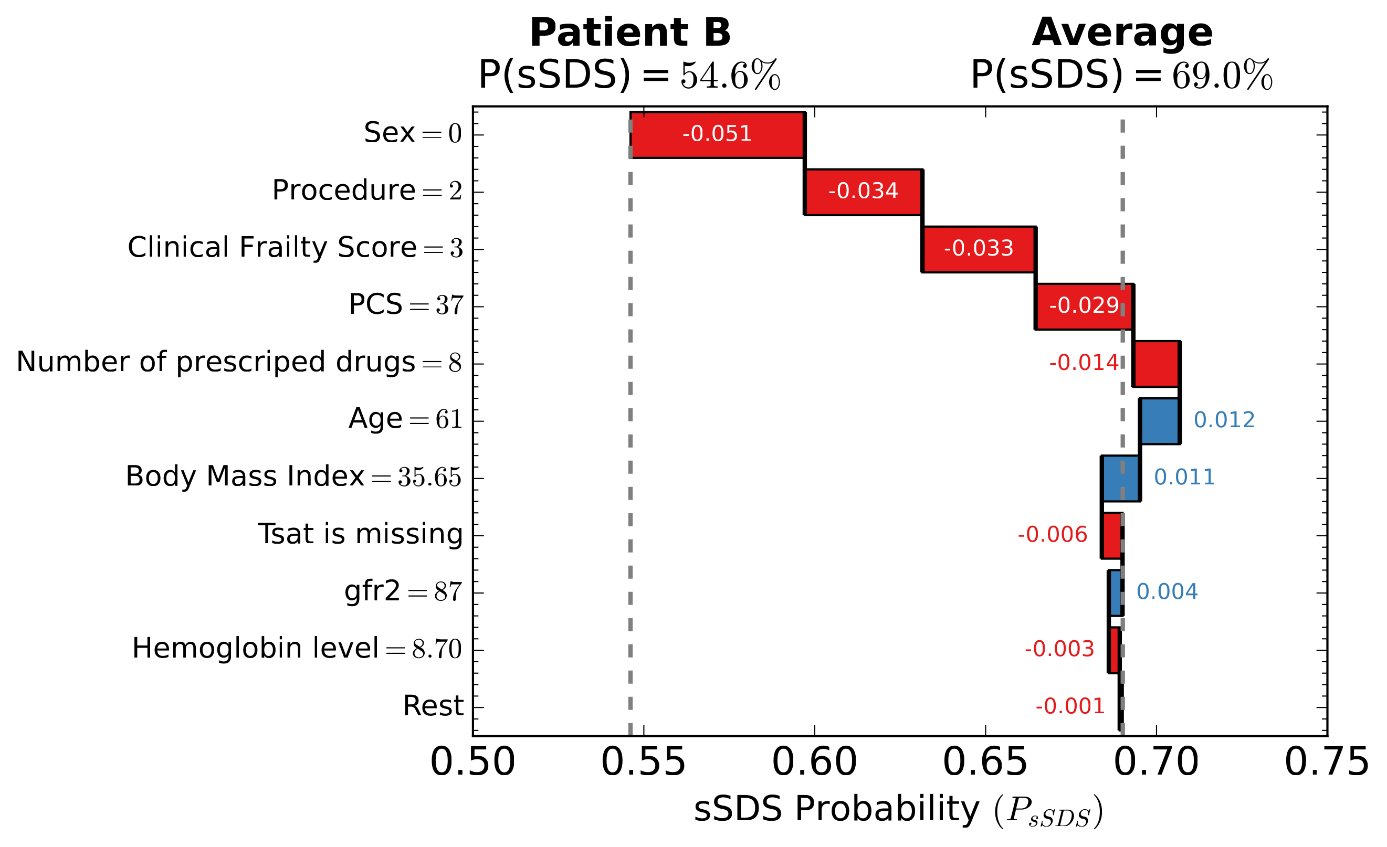


SHAP analysis of feature importances for probability of successful same-day surgery (sSDS) in a patient with a lower-than-average likelihood. Sex= 0 indicates female gender and Procedure= 2 indicates total knee arthroplasty. CFS: Clinical Frailty Score PCS: Pain catastrophizing score, Tsat: Transferrin saturation, gfr2 = glomerular filtration rate. Rest= Accumulated influence of remaining variables in the Common model. Dotted lines mark the calculated probability of sSDS in Patient B and the population average, respectively
